# Infections Requiring Hospitalization as Predictors of Pediatric-Onset Crohn's Disease and Ulcerative Colitis

**DOI:** 10.1155/2015/690581

**Published:** 2015-05-14

**Authors:** Susan Hutfless, Oren Abramson, Melvin B. Heyman, Theodore M. Bayless, De-Kun Li, Kevin Winthrop, Lisa J. Herrinton

**Affiliations:** ^1^Division of Gastroenterology & Hepatology, Johns Hopkins University, Baltimore, MD, USA; ^2^Department of Pediatrics, Kaiser Permanente Medical Center, Santa Clara, CA, USA; ^3^Department of Pediatrics, University of California, San Francisco, CA, USA; ^4^Division of Research, Kaiser Permanente Northern California, Oakland, CA, USA; ^5^Oregon Health & Science University, Portland, OR, USA

## Abstract

*Objectives.* To assess the relationship between infections and the risk of pediatric-onset inflammatory bowel disease (IBD).* Methods.* We conducted a nested case-control study of 501 incident cases aged ≤17 years and 9,442 controls who were members of Kaiser Permanente Northern California for at least one consecutive year between 1996 and 2006. IBD was confirmed and the incidence date was adjudicated by pediatric gastroenterologists. Hospitalized infections were identified from the principal diagnosis code of electronic inpatient records. Medications to treat infections were identified during the hospitalization. Conditional logistic regression was used to assess the associations between hospitalized infections, medications, and Crohn's disease and ulcerative colitis.* Results.* In the year prior to diagnosis, both hospitalized infection of any system (OR 6.3; 95% CI 1.6–23.9) and hospitalized intestinal infection (OR 19.4; 95% CI 2.6–143.2) were associated with CD. Hospitalized infections of any system were inversely associated with UC after excluding the year prior to diagnosis (OR 0.4; 95% CI 0.2–0.9). No UC case had a hospitalized gastrointestinal infection prior to diagnosis.* Conclusion.* Infections appear to play opposite roles prior to the diagnosis of CD and UC. Infections may be associated with an increased risk of CD and a decreased risk of UC.

## 1. Background

Crohn's disease (CD) and ulcerative colitis (UC) are putatively caused by a combination of genetic and environmental factors. Infections may act as an environmental trigger of CD and UC in a genetically predisposed individual by altering the intestinal microbiome or by affecting the development or response of the immune system. Infections may play a role in pediatric-onset disease initiation because of the rapid development of the immune system and intestinal microbiome that occurs during childhood [1].

Intestinal infections have been identified as a trigger of CD and UC among adults [2–5]. CD and UC are more common in the year after an intestinal infection compared with later periods. The association may be the result of detection bias from repeated testing or visits by patients presenting with nonspecific intestinal symptoms prior to an IBD diagnosis [4]. The association may not be entirely due to detection bias, because the increased risk of IBD persists after excluding the time period immediately after infection.

In addition to the potential differences infections may have on initiating disease in children compared with adults, infections may have a different effect on CD than on UC initiation. This would be comparable to the differences seen in CD and UC with regard to genetic risk factors (more genes identified for CD than UC) [6], smoking (possibly harmful for CD and protective for UC) [7], and appendectomy (no role in CD and protective for UC) [8, 9].

We conducted a case-control study of 501 incident cases and 9,442 matched controls from the Kaiser Permanente Northern California health plan to evaluate the role of infections requiring hospitalization with pediatric-onset CD, UC, and indeterminate colitis (IND). Timing of the infection prior to disease diagnosis and type of infection were evaluated. Medications during the hospitalization and at the time of discharge were accounted for in the analyses.

## 2. Methods

This work was conducted under the approval of the Institutional Review Boards of the Kaiser Foundation Research Institute and the Johns Hopkins School of Medicine.

### 2.1. Study Population

Kaiser Permanente Northern California is a prepaid, integrated system providing both health insurance and health care to 30% of the residents of the San Francisco Bay Area, averaging more than three million members per year. Extensive computerized information has been available since 1996, with hospitalization information dating back to 1984. The membership broadly represents the underlying population with regard to race and socioeconomic status [10]. All children (aged 17 and under) who were members for at least one consecutive year between 1996 and 2006 were eligible for the study. All study subjects had to have at least one healthcare utilization in the two years prior to their diagnosis date to ensure that they were actively using their Kaiser Permanente membership, and they could be diagnosed at a Kaiser Permanente facility and their medical history could be obtained.

#### 2.1.1. Cases

Cases were initially identified by inpatient and outpatient International Classification for Diseases, Clinical Modification 9 (ICD-9-CM) codes for IBD (555 for CD and 556 for UC), and then confirmed by medical record review by the pediatric gastroenterologists of Kaiser Permanente Northern California [11]. The pediatric gastroenterologists assigned a diagnosis date based on the combination of imaging studies and symptoms. We excluded 21 children who were diagnosed within one year of their first membership, to exclude children who may have joined the health plan because of symptoms related to IBD. The final number of cases was 501.

#### 2.1.2. Controls

We selected up to 20 controls for each case. The matched case's IBD diagnosis date was defined as the index date for the control. We required the potential control to be a health plan member on the matched case's index date and we matched the potential control to the case on age on the index date. We further required the control to be a health plan member on the matched case's first membership date. Control selection used incidence density sampling [12]. Similar to cases, controls were required to have at least one utilization within two years prior to the index date and were required to be a member for at least one year prior to the index date.

### 2.2. Data Collection

#### 2.2.1. Hospitalized Infection Ascertainment

We focused the study on infections requiring hospitalization because they are more objective than total infections, including both ambulatory and hospitalized infections. A hospitalized infection was defined as an infection listed as the principal diagnosis during hospitalization for any duration. All available information was obtained up to the day prior to the index date from the inpatient data sources to create the variable* hospitalized infection*. If a code for IBD (555 or 556) was recorded during the hospitalization for infection and prior to the confirmed diagnosis, the hospitalization was not counted as infection-related.

We analyzed infections using two concepts related to timing of infection relative to the diagnosis date. First, because hospitalized infections occurring immediately prior to diagnosis may reflect a close temporal relationship between infection and disease initiation, we evaluated hospitalized infections occurring in the year prior to the index date. Second, because the IBD disease process starts before the diagnosis of disease is made and hospitalized infections occurring after the disease is present cannot alter the risk of disease, that is, to rule out reverse causation and detection bias, we conducted an additional set of analyses after excluding infection information in the one year prior to the index date.

#### 2.2.2. Hospitalized Infections of Interest

We ascertained hospitalized infections with a principal diagnosis code of 001-139 (infectious and parasitic diseases): 380.1 (infective otitis externa); 381.0-4 and 382 (acute and chronic otitis media); 460-466 (acute respiratory infections); 480-488, 770.0, and 770.18 (pneumonia and influenza); 567 (peritonitis and retroperitoneal infections); 590 (infection of kidney); 670 (major puerperal infection); 680-686 (infections of skin and subcutaneous tissue); 771 (infections specific to the perinatal period). We also specifically examined intestinal (001-009, 567) and respiratory infections (460-466, 480-488, 770.0, and 770.18) based on their associations with IBD in previously published studies.

#### 2.2.3. Potential Confounding Factors

In addition to the matching factors, we also considered sex, race/ethnicity, and medications to treat infections as potential confounding factors. Race/ethnicity information was obtained from patient or family member report, when available, or from the information recorded in the inpatient or outpatient medical history. Categories of medications to treat infection included aminoglycosides, cephalosporins, erythromycins and related macrolides, penicillins, quinolones, sulfonamides, tetracyclines, and miscellaneous antimicrobials. Use of these medications during the hospitalization and filled prescriptions during the hospitalization or on the date of discharge were categorized during the same time periods as infections.

#### 2.2.4. Analyses

Conditional logistic regression accounting for the matching factors age, date of diagnosis, and duration of membership was used for all analyses and summarized using odds ratios (OR) and 95% confidence intervals (CI). ORs greater than 1 suggest infections are associated with an increased risk of IBD, whereas ORs less than 1 suggest infections are protective for IBD. Sex, race/ethnicity (non-Hispanic white versus others, due to sample size limitations), and medication use were also accounted for in all analyses. We examined the relationship between hospitalized infections and CD, UC, and IND separately.

#### 2.2.5. Sensitivity Analyses

To estimate the robustness of our definitions of timing of infection and coding position for infection, we performed sensitivity analyses. To test the robustness of excluding one year prior to the diagnosis to rule out reverse causation and detection bias, we also examined the relationship between hospitalized infections excluding the two years prior to the index date. We also examined hospitalized infections occurring during the two years prior to the index date. Analyses defining hospitalized infections as identified from any diagnosis code (ICD-9-CM in the principal position or through position 11) instead of only the principal position were also performed. Finally, we examined infections occurring at different ages, as infection may play a different role in disease initiation depending on the developmental stage of the immune system.

## 3. Results

### 3.1. Demographics

Forty-six percent of IBD cases were female compared with 49% of controls (*P* = 0.20; Table 1). Cases were more likely to be non-Hispanic white compared with controls (*P* < 0.0001). A family history was reported in the chart review for 21% of CD, 17% of UC, and 3 of 27 (8%) IND cases. At diagnosis, 62% of CD cases had ileal involvement and 41% of UC cases had pancolitis. The percent of patients hospitalized for any reason (infection or other indications) after birth was similar between cases and controls (26.8% IBD; 26.4% controls).

### 3.2. Relationship of Hospitalized Infections with Crohn's Disease

**Figure 1:**
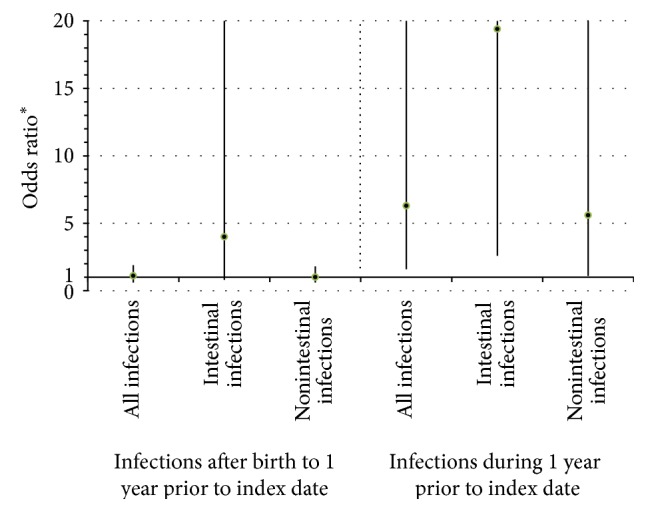
Odds ratios for hospitalized infections comparing Crohn's disease cases with controls. ^∗^Compared with controls using conditional logistic regression accounting for the matching factors: age and duration of membership as well as sex and race. Hospitalized infections were hospitalizations with diagnosis codes for infection in the principal diagnosis code position. Intestinal infections included ICD-9-CM codes 001-009 and 567. The index date was defined as the date of the case's diagnosis for both cases and matched controls.

**Figure 2:**
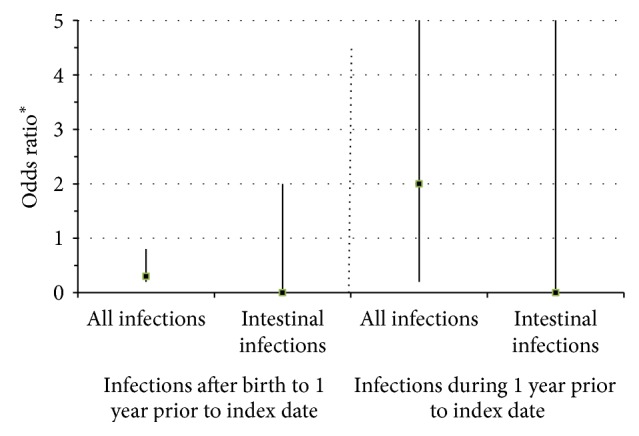
Odds ratios for hospitalized infections comparing ulcerative colitis cases with controls. ^∗^Compared with controls using conditional logistic regression accounting for the matching factors: age and duration of membership as well as sex and race. Hospitalized infections were hospitalizations with diagnosis codes for infection in the principal diagnosis code position. Intestinal infections included ICD-9-CM codes 001-009 and 567. The index date was defined as the date of the case's diagnosis for both cases and matched controls. Nonintestinal infections were not reported as no UC patient experienced an intestinal infection requiring hospitalization.

Hospitalized infections occurred in 6.9% of CD cases and 6.1% of controls excluding the year before diagnosis (OR 1.0; 95% CI 0.6–1.8; Table 2, Figures 1 and 2). During the year prior to diagnosis, hospitalized infections occurred in 1.4% of CD cases and 0.2% of controls. Hospitalized infections were associated with CD when examining the period one year prior to diagnosis (OR 6.3; 95% CI 1.6–23.9). All hospitalized cases received a medication precluding the inclusion of both variables in the same analysis.

The percents of hospitalized intestinal infections in CD cases and controls were 0.9% and 0.2% excluding the year before diagnosis and 0.9% and 0.1% during the year prior to diagnosis. Hospitalized infections of the intestinal system were associated with CD when examining the period one year prior to diagnosis (OR 19.4; 95% CI 2.6–143.2). This association persisted, although it was not statistically significant, when the year prior to diagnosis was excluded (OR 4.7; 95% CI 0.9–24.1). Examining nonintestinal infections alone produced similar results to the all infections analyses (Table 2). There was no meaningful difference between cases and controls for respiratory infections with none seen during the year prior to diagnosis among cases and fewer hospitalizations among cases than controls when the year prior to diagnosis was considered, albeit with wide confidence intervals (OR 0.7; 95% CI 0.2–2.1). Too few infections affecting other organ systems were observed to calculate ORs.

Four children were hospitalized multiple times (see supplementary Table 2 in Supplementary Material available online at http://dx.doi.org/10.1155/2015/690581). There was no clear trend in the diagnoses or medications used or laboratory tests among the children hospitalized multiple times.

### 3.3. Relationship of Hospitalized Infections and Ulcerative Colitis

Hospitalized infections occurred in 2.4% of UC cases and 6.1% of controls excluding the year before diagnosis (Table 2, Figures 1 and 2). Hospitalized infection was inversely associated with UC when the year prior to infection was excluded (OR 0.4; 95% CI 0.2–0.9). During the year prior to diagnosis, hospitalized infections occurred in 0.4% of UC cases and 0.2% of controls (OR 2.0; 95% CI 0.2–16.2). No UC case had a hospitalized intestinal infection prior to diagnosis. No UC case had a hospitalization for a respiratory infection in the year prior to diagnosis. All UC cases that were hospitalized received a medication precluding the inclusion of both variables in the same analysis. Too few infections affecting other organ systems were observed to calculate ORs.

Three children were hospitalized multiple times (Supplementary Table 3). There was no clear trend in the diagnoses and medications used or laboratory tests among the children hospitalized multiple times.

### 3.4. Indeterminate Colitis

Infections during the year prior to diagnosis were associated with an increased risk of disease, although not statistically significant (OR 10.0; 95% CI 0.9–113.8). The observed infection was respiratory, so that the respiratory infection OR is the same. Infections were not associated with IND when the infections during the year prior to diagnosis were excluded (OR 0.8; 95% CI 0.2–3.7), with similar respiratory infections OR (OR 0.8; 95% CI 0.1–5.7). No IND case had a hospitalized intestinal infection prior to diagnosis. No child was hospitalized more than one time prior to diagnosis (Supplementary Table 4).

### 3.5. Sensitivity Analyses

Hospitalized infections that occurred during the two years prior to the index date and, excluding this time period, had a similar trend to the one year threshold for CD, IND, and UC (data not shown). When we considered hospitalized infections defined as any diagnostic position rather than only the principal diagnosis position, minor differences were found (Supplementary Table 1). The CD recent nonintestinal infections became statistically significant after adjustment for medications and the UC recent infection associations adjusted for medications were estimable with results similar to the results that did not adjust for medications. The age at infection analyses did not produce meaningfully different findings from the main results (Supplementary Table 1).

## 4. Discussion

We observed an association of hospitalized infections with pediatric-onset CD. The finding was strongest for intestinal infections in the year preceding the diagnosis. Intestinal infections could be increasing the risk of CD but could also be a marker of undiagnosed disease. The nonstatistically significant elevation in intestinal infections when the year prior to diagnosis was excluded may suggest that infections may play a role in disease initiation in some patients. In contrast, we observed an inverse association of hospitalized infections with UC when the year prior to diagnosis was excluded. No hospitalizations for intestinal infections were observed among UC patients.

We observed that pediatric-onset CD cases were more likely to have had a hospitalization for an intestinal infection when we excluded the year prior to diagnosis, but we observed no intestinal infection hospitalizations among the children with UC. The stronger association of intestinal infections with CD than UC has been observed in other studies [13]. Other environmental risk factors that differ between CD and UC include cigarette smoke exposure and appendectomy [8, 9], both of which may affect disease by modifying or stimulating immunologic pathways.

Several possible hypotheses might explain the association of intestinal infections with CD. During the year prior to diagnosis, intestinal infections could be an indicator of undiagnosed disease [4] or play a causative role in triggering the disease, such as altering the intestinal microbiome [14]. Infections could also serve as a surrogate marker of antibiotic exposure and play no direct role in disease initiation. Several studies of adults and children have examined the role of antibiotic treatment as a risk factor for CD and UC, with antibiotic use consistently associated with an increased risk of disease [15–22]. In our study, after adjustment for medications used to treat infections, there were modest differences in the observed effect estimates. The lack of an observed relationship between nonintestinal infections and CD when the year prior to diagnosis was excluded (OR 0.9 after adjustment for medication use) may suggest that antibiotics to treat conditions outside of the gastrointestinal tract do not have the same impact on CD risk as intestinal infections and their treatments (OR 4.7 after adjustment for medications). The intestinal infection itself or the interaction of the intestinal infection with antibiotic treatment may play a stronger role in initiating CD than antibiotics for nonintestinal conditions.

The major limitation of this study of infections as a risk factor for IBD is the difficulty to distinguish a true infection from symptoms of IBD preceding diagnosis. This misclassification is more likely to affect CD where symptoms may precede diagnosis by months to years, in the form of gastrointestinal, peritoneal, and perianal symptoms that may be misclassified as infections, as opposed to UC where bloody diarrhea may lead to a more rapid diagnosis once enteric infections have been excluded. We have attempted to address this limitation by examining the relationship between intestinal infections and infections other than the intestinal ones, excluding infections in the year preceding diagnosis, and a sensitivity analysis in which infections were excluded in the 2 years prior to IBD diagnosis. According to the chart review, the mean time between symptoms and diagnosis was 6 months.

A second limitation of this study is the restriction to infections requiring hospitalization, excluding infections treated in an outpatient setting or managed without medical advice. Hospitalization for infection represents a serious infection that perhaps is less affected by care-seeking behavior than those infections seen in the outpatient clinic alone or not brought to professional medical attention. These serious infections could affect the immune system and intestinal microbiome more than an infection treated as an outpatient and thus could have greater impact on the risk of disease than a less severe infection. Examining infections occurring in inpatient and outpatient and settings without medical care would be required to robustly examine the hygiene hypothesis. The third limitation was the absence of medication information prior to 1995. All children hospitalized prior to 1995 were considered unexposed. In the supplementary tables, we list out all medications of interest taken by each child 1995 and later.

In conclusion, we found differences in the associations between hospitalized infections and the risk of CD and UC. Infections appear to play a different role in the initiation of pediatric onset compared with adult-onset inflammatory bowel disease. Factors that affect the development of the immune system and its composition may interact with infections to modify the risk of inflammatory bowel disease. Medications to treat infections do not meaningfully alter the observed relationships.

## Supplementary Material

Supplementary Files include the infection-disease associations considering infections in any diagnosis position (Table 1), the observed infections and medications taken during hospitalizations for the cases hospitalized for infections for Crohn's disease (Table 2), ulcerative colitis (Table 3) and indeterminate colitis (Table 4).

## Figures and Tables

**Table 1:** Demographics of cases and age and membership matched controls.

	Crohn's disease	Ulcerative colitis	Indeterminate colitis	All IBD	Controls^∗^
Number	216	248	37	501	9,442
Gender, %					
Female	44.4	48.8	37.8	46.1	49.1
Race, %					
Non-Hispanic white	65.7	63.3	43.2	62.9	47.5
Non-Hispanic African-American	10.7	5.2	10.8	8.0	7.3
Hispanic	7.9	16.5	13.5	12.6	14.8
Asian/Pacific Islander	2.8	15.7	13.5	5.0	11.6
Native American	0.5	0.4	0	0.4	0.4
Multiracial	7.4	4.4	10.8	6.2	3.0
Unknown	5.1	4.4	8.1	5.0	15.3
Age at index date, %					
0–4	3.7	4.8	10.8	4.8	4.9
5–9	12.0	10.1	18.9	11.6	11.8
10–14	42.6	41.9	37.8	41.9	41.1
15–17	41.7	43.2	32.4	41.7	42.3

IBD, inflammatory bowel disease.

^∗^Controls were matched to cases on age, sex, and length of enrollment.

The index date was defined as the date of the case's IBD diagnosis for both cases and matched controls.

**Table 2:** Adjusted odds ratio and 95% confidence intervals for the association of hospitalized infections with risk of Crohn's disease and ulcerative colitis by timing and type of infection.

Timing of infection	Type of infection^∗^	Controls, %	Cases, %	Adjusted OR^∗∗^	Antibiotics adjusted OR^∗∗∗^
				95% CI	95% CI
Crohn's disease					

Recent	All	0.2	1.4	6.3 (1.6–23.9)	NE^†^
	Nonintestinal	0.2	0.9	5.6 (1.1–28.2)	7.2 (0.7–69.8)
	Intestinal	0.1	0.9	19.4 (2.6–143.2)	19.4 (2.6–143.2)
	Respiratory	0.1	0	NE	NE

Distant	All	6.1	6.9	1.1 (0.6–1.9)	1.0 (0.6–1.8)
	Nonintestinal	6.0	6.5	1.0 (0.6–1.8)	0.9 (0.5–1.7)
	Intestinal	0.2	0.9	4.0 (0.8–20.0)	4.7 (0.9–24.1)
	Respiratory	2.6	1.9	0.7 (0.3–1.9)	0.7 (0.2–2.1)

Ulcerative colitis					

Recent	All	0.2	0.4	2.0 (0.2–16.2)	NE^†^
	Nonintestinal	0.2	0.4	2.0 (0.2–16.2)	NE^†^
	Intestinal	0.02	0	NE	NE
	Respiratory	0.02	0	NE	NE

Distant	All	6.1	2.4	0.3 (0.2–0.8)	0.4 (0.2–0.9)
	Nonintestinal	5.7	2.4	0.3 (0.2–0.8)	0.4 (0.2–1.0)
	Intestinal	0.4	0	NE	NE
	Respiratory	2.4	1.2	0.5 (0.1–1.5)	0.6 (0.2–1.9)

OR, odds ratio; NE, not estimable.

Recent = during the year before the index date.

Distant = after birth to one year before the index date.

^∗^Hospitalized infections were hospitalizations with diagnosis codes for infection in the principal diagnosis code position. Intestinal infections included ICD-9-CM codes 001-009 and 567. Children could contribute to the intestinal and nonintestinal infections analyses if they were hospitalized more than once. Respiratory infections included ICD-9-CM codes 460-466, 480-488, 770.0, and 770.18.

^∗∗^Compared with controls using conditional logistic regression accounting for the matching factors: age and duration of membership as well as sex and race.

^∗∗∗^Compared with controls using conditional logistic regression accounting for the matching factors: age and duration of membership as well as sex, race, and the use of medication in the following therapeutic classes during the hospitalization or at discharge: aminoglycosides, cephalosporins, erythromycins/related macrolides, miscellaneous antimicrobials, penicillins, quinolones, sulfonamides, or tetracyclines.

^†^NE, not estimable because all hospitalized cases received medication.
