# Supplementary material for: Infections Requiring Hospitalization as Predictors of Pediatric-Onset Crohn's Disease and Ulcerative Colitis
Source: Gastroenterol Res Pract. 2015 May 14;2015:690581. doi: 10.1155/2015/690581 (PMC4446489; doi:10.1155/2015/690581)
Supplement: Supplementary file 1 — Supplementary Files include the infection-disease associations considering infections in any diagnosis position (Table 1), the observed infections and medications taken during hospitalizations for the cases hospitalized for infections for Crohn's disease (Table 2), ulcerative colitis (Table 3) and indeterminate colitis (Table 4). [file 690581.f1.pdf]

Title: Infections Requiring Hospitalization as Predictors of Pediatric-Onset Crohn's Disease and Ulcerative Colitis

Authors: Susan Hutfless,[1] Oren Abramson,[2] Melvin B. Heyman,[3] Theodore M. Bayless,[1] De-Kun Li,[4] Kevin Winthrop,[5] Lisa J. Herrinton[4]

Table 1. Demographics of cases and age and membership matched controls

	Crohn's Disease	Ulcerative Colitis	Indeterminate Colitis	All IBD	Controls*
**Number**	216	248	37	501	9,442
**Gender, %**					
Female	44.4	48.8	37.8	46.1	49.1
**Race, %**					
Non-Hispanic White	65.7	63.3	43.2	62.9	47.5
Non-Hispanic African-American	10.7	5.2	10.8	8.0	7.3
Hispanic	7.9	16.5	13.5	12.6	14.8
Asian/Pacific Islander	2.8	15.7	13.5	5.0	11.6
Native American	0.5	0.4	0	0.4	0.4
Multiracial	7.4	4.4	10.8	6.2	3.0
Unknown	5.1	4.4	8.1	5.0	15.3
**Age at index date, %**					
0-4	3.7	4.8	10.8	4.8	4.9
5-9	12.0	10.1	18.9	11.6	11.8
10-14	42.6	41.9	37.8	41.9	41.1
15-17	41.7	43.2	32.4	41.7	42.3

IBD, inflammatory bowel disease
*Controls were matched to cases on age, sex and length of enrollment.
The index date was defined as the date of the case's IBD diagnosis for both cases and matched controls.

Table 2. Adjusted odds ratio and 95% confidence intervals for the association of hospitalized infections with risk of Crohn's disease and ulcerative colitis, by timing and type of infection.

Timing of infection	Type of infection*	Controls, %	Cases, %	Adjusted OR** 95% CI	Antibiotics adjusted OR*** 95% CI
**CROHN'S DISEASE**					
Recent	All	0.2	1.4	6.3 (1.6 – 23.9)	NE†
	Non-intestinal	0.2	0.9	5.6 (1.1 – 28.2)	7.2 (0.7 – 69.8)
	Intestinal	0.1	0.9	19.4 (2.6 – 143.2)	19.4 (2.6 – 143.2)
	Respiratory	0.1	0	NE	NE
Distant	All	6.1	6.9	1.1 (0.6 – 1.9)	1.0 (0.6 – 1.8)
	Non-intestinal	6.0	6.5	1.0 (0.6 – 1.8)	0.9 (0.5 – 1.7)
	Intestinal	0.2	0.9	4.0 (0.8 – 20.0)	4.7 (0.9 – 24.1)
	Respiratory	2.6	1.9	0.7 (0.3 – 1.9)	0.7 (0.2 – 2.1)
**ULCERATIVE COLITIS**					
Recent	All	0.2	0.4	2.0 (0.2 – 16.2)	NE†
	Non-intestinal	0.2	0.4	2.0 (0.2 – 16.2)	NE†
	Intestinal	0.02	0	NE	NE
	Respiratory	0.02	0	NE	NE
Distant	All	6.1	2.4	0.3 (0.2 – 0.8)	0.4 (0.2 – 0.9)
	Non-intestinal	5.7	2.4	0.3 (0.2 – 0.8)	0.4 (0.2 – 1.0)
	Intestinal	0.4	0	NE	NE
	Respiratory	2.4	1.2	0.5 (0.1 – 1.5)	0.6 (0.2 – 1.9)

OR, Odds Ratio; NE, Not estimable
Recent = During the year before the index date
Distant = After birth to one year before the index date
*Hospitalized infections were hospitalizations with diagnosis codes for infection in the principal diagnosis code position. Intestinal infections included ICD-9-CM codes 001-009 and 567. Children could contribute to the intestinal and non-intestinal infections analyses if they were hospitalized more than once. Respiratory infections included ICD-9-CM codes 460-466, 480-488, 770.0, and 770.18.
**Compared with controls using conditional logistic regression accounting for the matching factors age and duration of membership as well as sex and race.
***Compared with controls using conditional logistic regression accounting for the matching factors age and duration of membership as well as sex, race and the use of medication in the following therapeutic classes during the hospitalization or at discharge: aminoglycosides, cephalosporins, erythromycins/related macrolides, miscellaneous antimicrobials, penicillins, quinolones, sulfonamides, or tetracyclines.
† NE, Not estimable because all hospitalized cases received a medication

Supplemental Table 1. Adjusted odds ratio and 95% confidence intervals for the association of hospitalized infections, indicated in any diagnostic position, with risk of Crohn's disease and ulcerative colitis, by age, timing and type of infection.

Timing of infection	Type of infection*	Controls, %	Cases, %	Adjusted OR** 95% CI	Antibiotics adjusted OR*** 95% CI
			**CROHN'S DISEASE**		
**Age 0 – 5**					
Recent	All	0.6	5.9	9.3 (1.7 – 51.6)	9.7 (0.9 – 109.0)
	Non-intestinal	0.5	5.9	13.5 (2.2 – 82.4)	22.6 (3.1 – 165.4)
	Intestinal	0.2	2.9	20.8 (1.2- 360.6)	NE
	Respiratory	0.2	0	NE	NE
Distant	All	9.4	5.9	0.6 (0.1 – 2.7)	0.8 (0.2 – 3.6)
	Non-intestinal	9.4	5.9	0.6 (0.1 – 2.7)	0.8 (0.2 – 3.6)
	Intestinal	0	0	NE	NE
	Respiratory	4.5	2.9	0.7 (0.1 – 5.5)	1.1 (0.1 – 8.9)
**Age 10 – 17**					
Recent	All	0.3	2.2	8.0 (2.4 – 26.7)	5.2 (0.5 – 50.5)
	Non-intestinal	0.2	1.7	6.7 (1.7 – 26.2)	5.2 (0.5 – 50.7)
	Intestinal	0.03	0.6	18.5 (1.1 – 306.8)	NE
	Respiratory	0.03	0	NE	NE
Distant	All	8.3	7.7	0.9 (0.5 – 1.5)	0.8 (0.4 – 1.5)
	Non-intestinal	8.2	7.1	0.8 (0.4 – 1.4)	0.7 (0.4 – 1.4)
	Intestinal	0.2	1.1	4.0 (0.8 – 20.1)	4.7 (0.9 – 24.1)
	Respiratory	3.4	2.2	0.6 (0.2 – 1.7)	0.6 (0.2 – 1.8)
			**ULCERATIVE COLITIS**		
**Age 0 – 5**					
Recent	All	0.7	0	NE	NE
	Non-intestinal	0.6	0	NE	NE
	Intestinal	0.1	0	NE	NE
	Respiratory	0.3	0	NE	NE
Distant	All	9.1	8.1	0.9 (0.3 – 2.9)	0.7 (0.2 – 3.2)
	Non-intestinal	8.8	8.1	0.9 (0.3 – 2.9)	0.7 (0.2 – 3.3)
	Intestinal	0.4	0	NE	NE
	Respiratory	4.4	5.4	1.2 (0.3 – 5.3)	1.7 (0.4 – 7.5)

Formatted Table

Timing of infection	Type of infection*	Controls, %	Cases, %	Adjusted OR** 95% CI	Antibiotics adjusted OR*** 95% CI
**Age 10 – 17**					
Recent	All	0.3	1.0	3.1 (0.7 – 14.2)	3.9 (0.5 – 33.9)
	Non-intestinal	0.3	1.0	3.1 (0.7 – 14.2)	3.9 (0.5 – 33.9)
	Intestinal	0	0	NE	NE
	Respiratory	0.07	0.5	5.9 (0.6 – 58.7)	NE
Distant	All	7.6	4.3	0.5 (0.2 – 1.0)	0.6 (0.3 – 1.2)
	Non-intestinal	7.3	4.3	0.5 (0.3 – 1.0)	0.6 (0.3 – 1.2)
	Intestinal	0.4	0	NE	NE
	Respiratory	2.9	1.4	0.4 (0.1 – 1.5)	0.6 (0.2 – 1.9)

OR, Odds Ratio; NE, Not estimable
Recent = During the year before the index date
Distant = After birth to one year before the index date
*Hospitalized infections were hospitalizations with diagnosis codes for infection in the principal diagnosis code position. Intestinal infections included ICD-9-CM codes 001-009 and 567. Children could contribute to the intestinal and non-intestinal infections analyses if they were hospitalized more than once.
**Compared with controls using conditional logistic regression accounting for the matching factors age and duration of membership as well as sex and race.
***Compared with controls using conditional logistic regression accounting for the matching factors age and duration of membership as well as sex, race and the use of medication in the following therapeutic classes during the hospitalization or at discharge: aminoglycosides, cephalosporins, erythromycins/related macrolides, miscellaneous antimicrobials, penicillins, quinolones, sulfonamides, or tetracyclines.
~~† NE, Not estimable because all hospitalized controls received a medication (75% of cases)~~
~~†† NE, Not estimable because no hospitalized cases received a medication~~

Formatted Table

Supplement Table 2. Hospitalized infections observed in Crohn's disease cases prior to diagnosis

Case	Race	IBD type	Time between first symptoms and diagnosis (years)	Age of diagnosis	Age infection (years)	Time between infection and dx	Principal diagnosis	Type of infection	Medications during hospitalization*	Specific pathogen laboratory results?*	Length of stay (days)
Female #1	White	CD	3.5	4	6 months	3.3 years	Other unspecified non-infectious gastroenteritis and colitis	Acute upper respiratory infection, unspecified site	None	None	<1
Female #2	White	CD	0.9	5	5	11 days	Abdominal pain	Intestinal infections due to other organisms	Metronidazole, Ampicillin, Gentamicin	None	4
Female #3	White	CD	0.2	8	1	6.5 years	Otitis media	Otitis media	<1995	<1995	13
Female #4	White	CD	0.6	12	12	18 days	Anal fistula	Cellulites or abscess on trunk	None	None	<1
Female #5	White	CD	0.0	13	9 months	12.1 years	Acute pyelonephritis	Streptococcus	<1995	<1995	2
Female #6	White	CD	0.1	14	14	11 days	Blood in stool	Colitis, enteritis, and gastroenteritis of presumed infectious origin	Ceftriaxone, Quinolones (unspecified)	Clostridium Difficile toxin negative. No E coli O157:H7, Salmonella, Shigella, or Campylobacter isolated	2
Female #7	Multiple races	CD	0.1	17	1	16 years	Intestinal infection due to other organism, not elsewhere classified	Intestinal infection due to other organism, not elsewhere classified	<1995	<1995	3
Female #8	White	CD	0	17	1	16 years	Otitis media	Otitis media	<1995	<1995	<1
Female #9, Hospitalization #1	White	CD	0	17	1 month	17 years	Unspecified viral infection	Unspecified viral meningitis	<1995	<1995	3

**Case**	**Race**	**IBD type**	**Time between first symptoms and diagnosis (years)**	**Age of diagnosis**	**Age infection (years)**	**Time between infection and dx**	**Principal diagnosis**	**Type of infection**	**Medications during hospitalization***	**Specific pathogen laboratory results?***	**Length of stay (days)**
Female #9, Hospitalization #2					8	9 years	Cellulitis or abscess on face	Cellulitis or abscess on face and Methicillin susceptible Staphylococcus aureus	Cephalosporins (unspecified)	None	1
Female #10	White	CD	0.4	17	2	16 years	Otitis media	Otitis media	<1995	<1995	<1
Male #1, Hospitalization #1	Multiracial	CD	0.3	3	3	4 months	Unspecified viral infection	Unspecified viral infection	None	None	4
Male #1, Hospitalization #2					3	7 days	Clostridium difficile	Clostridium difficile	Vancomycin	None	2
Male #2	White	CD	0.3	10	3	7 years	Otitis media	Otitis media	None	None	<1
Male #3	White	CD	0.2	11	9 months	10 years	Cellulitis or abscess of hand	Cellulitis or abscess of hand and Methicillin susceptible Staphylococcus aureus and Candidiasis of skin or nails	Nafcillin	None	3
Male #4, Hospitalization #1	White	CD	0.1	11	1 month	11 years	Extreme immaturity	Other infection specific to the perinatal period and impetigo	<1995	<1995	103
Male #4, Hospitalization #2				11	5 months	11 years	Infection or inflammatory reaction due to vascular device, implant or graft	Other specified septicemia	<1995	<1995	19
Male #4, Hospitalization #3				11	6 months	11 years	Other suppurative peritonitis	Other suppurative peritonitis and Candidiasis of mouth	<1995	<1995	39

Case	Race	IBD type	Time between first symptoms and diagnosis (years)	Age of diagnosis	Age infection (years)	Time between infection and dx	Principal diagnosis	Type of infection	Medications during hospitalization*	Specific pathogen laboratory results?*	Length of stay (days)
Male #4, Hospitalization #4				11	6 months	11 years	Other or unspecified postsurgical intestinal malabsorption	Candidiasis of mouth and acute bronchiolitis and otitis media	<1995	<1995	41
Male #4, Hospitalization #5				11	1	10 years	Other or unspecified postsurgical intestinal malabsorption	Candidiasis of skin and nails	<1995	<1995	38
Male #4, Hospitalization #6				11	1	10 years	Colostomy and enterostomy complication	Other specified bacterial infection	<1995	<1995	6
Male #4, Hospitalization #7				11	1	10 years	Other or unspecified postsurgical intestinal malabsorption	Acute upper respiratory infection and unspecified viral or chlamydial infection	<1995	<1995	122
Male #4, Hospitalization #8				11	9	2.5 years	Pneumonia	Pneumonia	Erythromycins/related macrolides (unspecified)	None	2
Male #4, Hospitalization #9				11	11	4 months	Unspecified viral infection	Unspecified viral infection	Metronidazole, Ampicillin, Gentamicin	C difficile toxin negative; No salmonella, shigella or campylobacter isolated	2
Male #5	White	CD	0.1	12	1	10 years	Otitis media	Otitis media	<1995	<1995	<1
Male #6	White	CD	0	13	6	7 years	Otitis media	Otitis media	None	None	<1
Male #7	White	CD	2.2	13	2 months	13 years	Otitis media	Otitis media	<1995	<1995	2
Male #8, Hospitalization #1	Hispanic	CD	0.2	15	6	9 years	Pneumonia	Pneumonia	<1995	<1995	1

Case	Race	IBD type	Time between first symptoms and diagnosis (years)	Age of diagnosis	Age infection (years)	Time between infection and dx	Principal diagnosis	Type of infection	Medications during hospitalization*	Specific pathogen laboratory results?*	Length of stay (days)
Male #8, Hospitalization #2					9	6 years	Pneumonia	Pneumonia	<1995	<1995	3
Male #9	White	CD	0	15	6	9 years	Acute tracheitis without mention of obstruction	Acute tracheitis without mention of obstruction and pneumonia due to Staphylococcus	<1995	<1995	7
Male #10	White	CD	0	15	4 months	15 years	Acute bronchitis	Candidiasis of mouth	<1995	<1995	2
Male #11	White	CD	0.2	17	17	2 months	Suppurative peritonitis	Suppurative peritonitis	Metronidazole, Ciprofloxacin, Cefotetan, Gentamicin	None	8

*Pharmacy and laboratory reports available 1995 and later. <1995 indicates that hospitalization occurred prior to 1995 when information was not available.

Supplement Table 3. Hospitalized infections observed in ulcerative colitis cases prior to diagnosis

Case # (to identify multiple infections in same kid)	Race	IBD type	Time between symptoms and diagnosis (years)	Age of diagnosis (years)	Age infection (years)	Time between infection and dx (years)	Principal diagnosis	Type of infection	Medications during hospitalization*	Specific pathogen laboratory results?*	Length of stay (days)
Female #1	White	UC	0	5	0	5	Birth	Congenital pneumonia	None	None	<1
Female #2	White	UC	0.1	5	1.1	4.0	Croup	Croup	None	None	<1
Female #3, Hospitalization #1	White	UC	0	10	4.1	6.3	Unspecified viral infection	Unspecified viral infection	<1995	<1995	2
Female #3, Hospitalization #2					6.3	4.0	Acute polyarticular juvenile rheumatoid arthritis	Acute lymphadenitis	None	No salmonella, shigella or campylobacter isolated; No yersinia enterocolitica isolated; Negative for clostridium difficile toxin; Infectious Mononucleosis Screen negative, EBV negative; Leptospira positive, CMV positive, Adenovirus positive	10
Female #4	White	UC	0.3	11	0.9	10	Convulsions	Unspecified viral or chlamydial infection	<1995	<1995	3

Case # (to identify multiple infections in same kid)	Race	IBD type	Time between symptoms and diagnosis (years)	Age of diagnosis (years)	Age infection (years)	Time between infection and dx (years)	Principal diagnosis	Type of infection	Medications during hospitalization*	Specific pathogen laboratory results?*	Length of stay (days)
Female #5	White	UC	0	13	6.3	6.4	Hypertrophy of tonsils with adenoids	Otitis media	<1995	<1995	1
Female #6, Hospitalization #1	White	UC	0	13	1.7	11.7	Otitis media	Otitis media	<1995	<1995	<1
Female #6, Hospitalization #2					3.1	10.3	Hypertrophy of adenoids	Otitis media	<1995	<1995	<1
Female #7	White	UC	0	15	2.4	12.7	Acute febrile mucocutaneous lymph node syndrome	Streptococcal sore throat	<1995	<1995	5
Female #8	Hispanic	UC	0	16	14.5	1.2	Pilonidal cyst with abscess	Pilonidal cyst with abscess	None	None	1
Male #1	White	UC	0.2	6	2.6	3.6	Extrinsic asthma with status asthmaticus	Otitis media	Erythromycin	None	3
Male #2	White	UC	0.1	10	9.6	0.4	Coats' syndrome	Acute pharyngitis	None	None	0
Male #3	White	UC	0	12	11.2	0.5	Acute lymphadenitis	Acute lymphadenitis	Ampicillin	Anaerobe culture negative	3
Male #4, Hospitalization #1	White	UC	0.2	16	1.6	14.1	Pneumonia	Pneumonia and otitis media	<1995	<1995	1
Male #4, Hospitalization #2					3.2	12.5	Chickenpox	Chickenpox and scarlet fever	<1995	<1995	2
Male #5	White	UC	0.3	16	4.7	11.7	Croup	Croup	<1995	<1995	1
Male #6	Hispanic	UC	0.1	17	5.5	11.4	Extrinsic asthma with status asthmaticus	Pneumonia	None	None	4

*Laboratory reports available 1995 and later. <1995 indicates that hospitalization occurred prior to 1995 when information was not available.

Supplement Table 4. Hospitalized infections observed in indeterminate colitis cases prior to diagnosis

Case # (to identify multiple infections in same kid)	Race	IBD type	Time between symptoms and diagnosis (years)	Age of diagnosis	Age infection	Time between infection and dx (years)	Principal diagnosis	Type of infection	Medications during hospitalization*	Specific pathogen laboratory results?*	Length of stay (days)
Female #1	Hispanic	IC	0	8	8.0	0.3	Acute tonsillitis	Acute tonsillitis	None	None	<1
Male #1	Asian	IC	0.2	7	0.4	6.9	Acute respiratory syncytial virus	Acute respiratory syncytial virus and pneumonia	Erythromycin, Cefaclor, Amoxicillin	No Bordetella isolated, bordetella direct fa negative; Positive for resp. syncytial virus direct fa	3
Male #2	Multiracial	IC	0.1	13.8	0.1	13.7	Acute pyelonephritis without lesion of renal medullary necrosis	Acute pyelonephritis without lesion of renal medullary necrosis and Escherichia coli and Candidiasis of skin and nails	<1995	<1995	4
Male #3	Multiracial	IC	0	17	4.5	13.4	Otitis media	Otitis media	<1995	<1995	<1

*Laboratory reports available 1995 and later. <1995 indicates that hospitalization occurred prior to 1995 when information was not available.
